# The Ethics of Human Brain Organoid Transplantation in Animals

**DOI:** 10.1007/s12152-023-09532-3

**Published:** 2023-10-04

**Authors:** Masanori Kataoka, Christopher Gyngell, Julian Savulescu, Tsutomu Sawai

**Affiliations:** 1 Graduate School of Humanities and Social Sciences, Hiroshima University, Higashi-Hiroshima, Japan; 2 Biomedical Ethics Research Group, Murdoch Children’s Research Institute, Melbourne, Australia; 3 Department of Paediatrics, The University of Melbourne, Melbourne, Australia; 4 Faculty of Philosophy, The University of Oxford, Oxford, UK; 5 Melbourne Law School, The University of Melbourne, Melbourne, Australia; 6 Centre for Biomedical Ethics, Yong Loo Lin School of Medicine, National University of Singapore, Singapore, Singapore; 7 Institute for the Advanced Study of Human Biology (ASHBi), Kyoto University, Kyoto, Japan

**Keywords:** Brain organoids, Chimeras, Transplantation, Consciousness, Enhancement

## Abstract

In this paper, we outline how one might conduct a comprehensive ethical evaluation of human brain organoid transplantation in animals. Thus far, ethical concerns regarding this type of research have been assumed to be similar to those associated with other transplants of human cells in animals, and have therefore not received significant attention. The focus has been only on the welfare, moral status, or mental capacities of the host animal. However, the transplantation of human brain organoids introduces several new ethical issues. Many of these are related to uncertainty regarding whether or not brain organoids might be conscious. While these concerns might not be immediately relevant, they warrant closer scrutiny. We discuss how various ethical issues are relevant to different stages of human brain organoid transplantation and can guide the ethical evaluation of research. Our examination would broaden the horizons of the debate on the transplantation of brain organoids.

## Introduction

Since being first reported in 2018 [1], several transplantations of three-dimensional human brain tissues derived from pluripotent stem cells, called “brain organoids,” have been conducted in animals. Recently, one case received significant attention [2]. In this case, the transplanted brain organoids grew to occupy approximately one-third of the hemisphere of the host rat brain. Transplanted organoids were also integrated with neural circuits in the sensory cortex of the rat. It was further reported that optogenetic stimulation of the transplanted human brain organoids changed the behavior of the host rat.

To date, the ethical discussions in this study have focused on issues familiar to other bioethics areas, such as chimera research. In an interview with Sergiu Pasça, who led the study, concerns were raised about animal welfare, cognitive enhancement, consent, blurring of the “species boundaries,” and the possibility of brain organoids becoming conscious [3]. Pasça addressed the first two concerns, noting that the study did not observe any distress or enhancement in host rats. Bioethicist Insoo Hyun reported no health problems or cognitive enhancement in rats. He also mentioned that transplanted brain organoids were given more complex inputs and outputs than those *in vitro,* but he did not examine related ethical issues [4].

More generally, in a study on the ethical issues surrounding human brain organoid transplantation in animals, H. Isaac Chen and colleagues considered human brain organoid transplantation an example of general neurological chimera research [5].^1^ Similarly, a detailed ethical analysis of two cases of human brain organoid transplantation focused on issues related to chimeric animals produced *via* the implantation of brain organoids [7].^2^

However, to generate a comprehensive analysis of human brain organoid transplantation in animals, it is necessary to go beyond issues related to chimeric animals and consider the transplanted human brain organoids and their potential moral status. Although there is an active ethical debate around human brain organoids *in vitro*, few studies have examined transplanted human brain organoids in detail. Even these few exceptional studies focus only on a part of the research process (the stage when the transplanted human brain organoid is functionally fully integrated with the host brain) and fail to provide an overview of the various ethical issues that would arise throughout the research process [11–13].^3^^,^ 4

In this study, we provide a comprehensive step-by-step analysis of human brain organoid transplantation in animals. Two key objectives guided this analysis.

**Fig. 1:**
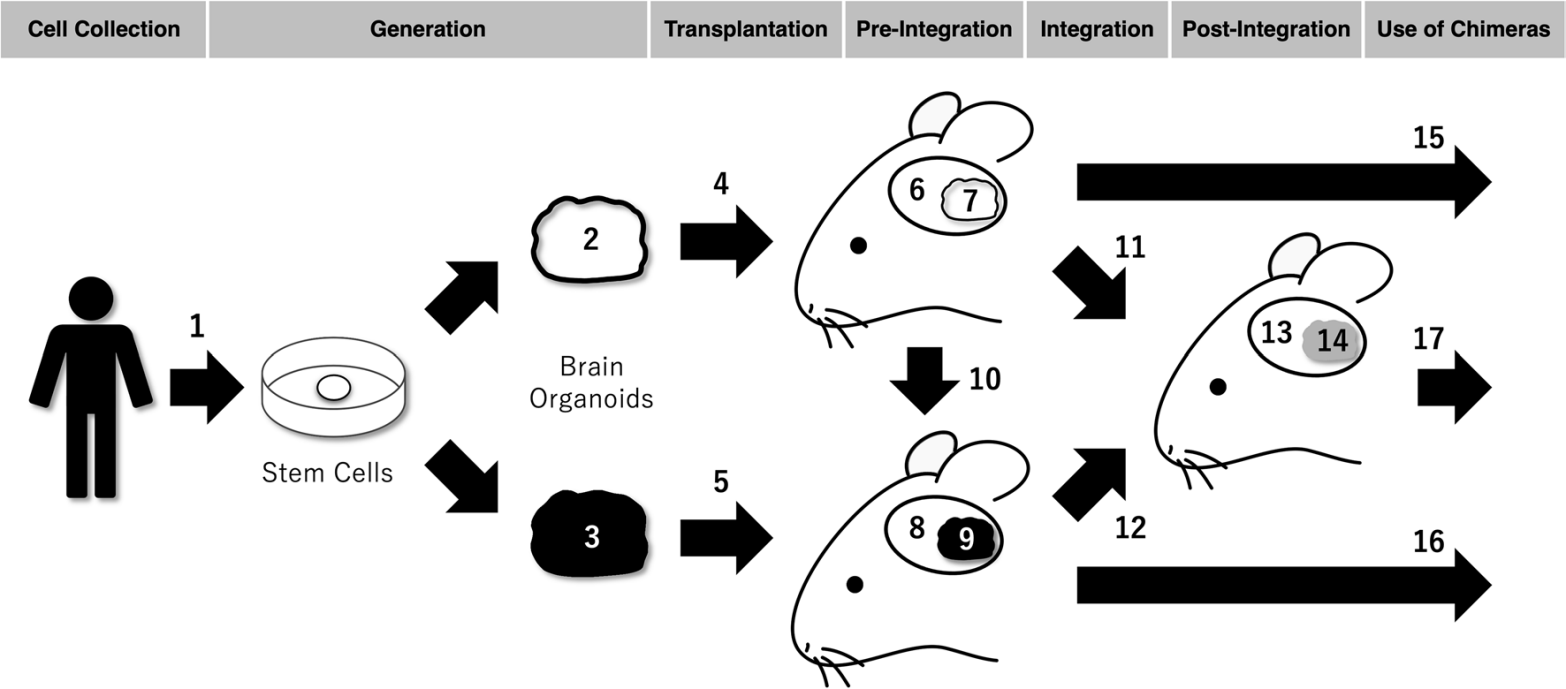
Research process of human brain organoid transplantation in animals. In the Cell Collection stage, somatic cells or embryos are obtained from donors (1). In the Generation stage, pluripotent stem cells are produced from donated cells, and brain organoids are generated. Brain organoids can be either non-conscious (2) or conscious (3) at the time of transplantation (non-conscious brain organoids are represented by white and conscious organoids by black). In the Transplantation stage, the non-conscious or conscious brain organoid is transplanted into the animal brain (4 or 5). The Pre-Integration stage is the stage in which the transplanted human brain organoid and the host brain are not functionally integrated. This stage can be divided into two cases: one in which the host brain (6) and non-conscious brain organoids (7) coexist, and the other in which the host brain (8) and conscious brain organoids (9) coexist. The former case may move to the latter as non-conscious brain organoids mature in the host brain and may become conscious (10). The Integration stage refers to the functional integration of the transplanted non-conscious/conscious brain organoid with the host brain (11 or 12). As a result, in the Post-Integration stage, the host brain (13) and the transplanted brain organoids (14) are functionally well-integrated. It is unclear whether the transplanted brain organoid can be an independent subject of consciousness (represented in gray). Finally, in the Use of Chimeras stage, chimeric animals in the Pre-Integration stage (15 or 16) or Post-Integration stage (17) are used in various ways for research purposes. Each number corresponds to the number in bold in the main text

1. To clarify the distinction between ethical issues related to chimeric animals and those related to transplanted human brain organoids. This highlights the new problems specific to human brain organoid transplantation in animals.
2. To identify and discuss ethical issues relevant to each stage in the research process (Fig. 1).

Some of the issues identified and examined below are speculative. Several issues specific to human brain organoid transplantation in animals are related to the “consciousness” of brain organoids. Here, we emphasize that it is unlikely that brain organoids are conscious, as per our current understanding (see below). Even the most primitive consciousness would require a fairly complex and extensive neural architecture, which brain organoids cannot recapitulate. Therefore, issues related to the consciousness of organoids are highly speculative and not of pressing importance. However, ethicists should focus not only on the most pressing issues but also on neglected issues that may arise in the future. Anticipatory considerations can guide future research, thereby preventing ethical problems from arising before it is too late. This is particularly true for the transplantation of human brain organoids into animals. Human brain organoid transplantation in animals is rapidly developing; however, only a few studies have examined the relevant ethical issues. We hope that our examination of these issues will guide future research in this area.

Before proceeding, we make some terminological notes. While it is often concerning that human brain organoids could have “consciousness,” this term is very general and can mean any of a variety of specific forms of consciousness. The following three forms have been discussed, particularly in relation to brain organoids. Namely, having non-valenced experience (i.e., experience without any feeling of good or bad), valenced experience or sentience (i.e., the capacity to experience a feeling of good or bad, such as pain or pleasure), and advanced cognitive functions, including self-awareness.^5^ In this paper, for simplicity, we continue to use the general term “ consciousness,” unless specified otherwise. Thus, the readers may understand “conscious” or “consciousness” to refer to any specific form of consciousness that they think is morally important. We will make it explicit if and when any specific form of consciousness is under discussion.

## Cell Collection

Consider the Cell Collection stage shown in Fig. 1. At this stage, human cells are collected to generate pluripotent stem cells (e.g., embryonic stem cells and induced pluripotent stem cells) (**1**), which are used to generate human brain organoids for transplantation. Among the well-known problems with the acquisition and use of pluripotent stem cells (especially embryonic stem cells), obtaining consent from donors of embryonic or somatic cells is particularly important in the context of human brain organoid transplantation into animals [21, 22].

The creation of chimeric animals and human brain organoids is a morally sensitive issue for donors respectively. Thus, using donor cells for generating brain organoids, which are then implanted into animal brains, is a doubly sensitive and controversial application. While debate continues regarding the consent method for such sensitive research, blanket or broad consent does not seem appropriate [23]. When informing cell donors of the nature of the research, ethical issues related to the transplantation of human brain organoids should be communicated explicitly.

Nevertheless, some individuals are willing to donate their cells for ethically controversial research, which is likely true for human brain organoid transplantation in animals. Even if only 5% of possible donors agreed to allow their cells to be used for brain organoid transplant research, many brain organoids could be created for this purpose.

## Generation

The next stage involves the *in vitro* generation of human brain organoids from pluripotent stem cells. There is active debate on whether and when brain organoids *in vitro* could achieve consciousness, partly because much depends on which theory of consciousness one adopts [14]. While there is a consensus among scientists that, in the current and probable future, brain organoids are unlikely to be sophisticated enough to have consciousness [8], this may change as technology and theories of consciousness develop. For ethical analysis, we assume that it will be possible to create conscious brain organoids in the future.^6^

For a comprehensive ethical analysis, it is important to distinguish between cases in which the brain organoids used for transplantation are non-conscious (**2**) and those in which they are conscious (**3**). Many concerns about creating conscious brain organoids *in vitro* have been voiced, and we shall not repeat these points here (see note 5). However, we briefly make a point that is particularly relevant to transplantation. Brain organoids transplanted into animals are more likely to be region-specific rather than recapitulating the brain as a whole by self-patterning. Thus, if a broad neural network is necessary to realize consciousness, it is unlikely that conscious brain organoids would be created during the Generation stage.^7^

That said, concerns regarding the creation of conscious organoids *in vitro* are not unrelated to transplantation. Consider the possibility that *in vitro* non-conscious brain organoids are transplanted into host animals and become conscious *in vivo* (**9**). The *in vivo* creation of conscious brain organoids raises almost identical ethical concerns, as would be the case if they were created *in vitro*. We address this point in "Conscious Human Brain Organoids in vivo".

## Transplantation

### Transplantation in General and of Non-conscious Human Brain Organoids

Transplantation of human brain organoids into animal brains (**4**, **5**) results in the creation of chimeric animals. Creating such human-animal chimeras is often called the “biological humanization” of animals [24]. Biological humanization is contrary to the common (but problematic) belief that “species boundaries” are fixed, and has raised various concerns, including threats to common beliefs [25] and impingement on human dignity [26].

On the one hand, many ethicists do not consider biological humanization *per se* to be morally problematic [27]. What they consider important is that chimeric animals may acquire a moral status similar to that of humans by gaining, for example, enhanced cognitive and evaluative functions through biological humanization. This is often referred to as “moral humanization” [24].^8^ We discuss this point further in later stages (**6**, **8**, **13**), because it concerns the status of chimeric animals rather than the act of transplanting human brain organoids.

On the other hand, the general public may have concerns about biological humanization. This should not be ignored in context of regulating such research. The regulation of research is more likely to be accepted and engender trust in society if it considers views of ordinary citizens. To date, public attitudes toward animal transplantation of human brain organoids have not been sufficiently examined. There is only one relevant study, a small interview with US citizens, in which the majority of participants were in favor of animal transplantation of human brain organoids, although some were concerned about blending humans and animal elements [29].^9^ Until citizens’ perspectives on human brain organoid transplantation are sufficiently reviewed, we will not be able to discuss this issue further. However, at least for neurological chimeras by blastocyst complementation, a survey showed that approximately half of the US citizens see their creation as unacceptable [31]. There could potentially be many opponents of animal brain-organoid transplants as well. Of course, social surveys are not the only way to engage the public. Bidirectional and inclusive public engagement through various methods will become increasingly important in the research and governance of brain organoid transplantation [32].

Human brain organoids used for transplantation may or may not be conscious *in vitro*. This difference has significant implications for the evaluation of ethical issues during the Transplantation stage. Indeed, the ethical issues in the act of transplanting nonconscious human brain organoids into animal brains (**4**) do not appear to differ from those in the act of transplanting other human cells into animal brains. In both cases, biological humanization occurs.^10^ However, transplanting liposomes from human donors into animals, for example, if useful for research purposes, is permitted under many research ethics standards. Thus, in principle, the same can be said about the transplantation of nonconscious human brain organoids, at least at the Transplantation stage.

### Transplantation of Conscious Human Brain Organoids

Future transplantations may use more sophisticated human brain organoids. This is plausible, given that the potential medical application of human brain organoids is their transplantation into the *human* brain to repair brain damage caused by stroke or an accident. To repair highly structured brain regions, such as the cortex and hippocampus, some consider using brain organoids of the corresponding areas as effective because they are similarly structured [33, 34]. To develop these techniques, sophisticated human brain organoids may need to be transplanted into animal brains at some stage. It may be possible that the type of brain organoid required for this application is conscious.

Are there unique ethical problems associated with the transplantation of conscious human brain organoids into animals (**5**)? Andrea Lavazza makes the following argument [14]: human brain organoids with sentience (i.e., the capacity to feel pain and pleasure) should be granted a moral status. For Lavazza, the transplantation of human brain organoids into animals is analogous to cases involving human subjects connected to a host entity such that they cannot live without the host, similar to the famous Thomson’s violinist thought experiment with regard to the abortion debate [35].^11^ Lavazza considered this a gross violation of human dignity. Similarly, he states that this is true for the transplantation of a sentient human brain organoid into animals. In this case, the dignity of the transplanted human organoids is violated. Therefore, Lavazza concluded that transplanting sentient human brain organoids into animals is highly problematic.

There are objections to giving sentient human brain organoids the same moral status as that of humans. Some ethicists have compared the moral status of sentient human brain organoids with that of sentient animals [16]. If this is the case, how problematic would the transplantation of sentient human brain organoids into animals be?

To address this point, it may be helpful to consider how similar experiments on animals are ethically evaluated and then those considerations may be applied to human brain organoid transplantation. How should one ethically evaluate the connection of an animal to a host entity so that it cannot live without the host? Perhaps the closest example of such an experiment is head transplantation in mice. Some researchers claim to have created a mouse with two active heads by transplanting the head of one mouse into another [36]; however, the scientific validity of such head transplantation has been questioned [37].

Given that this experiment can cause great harm to donor mice and that mice have some moral status, it would be unethical unless there is some convincing justification according to the standard ethical guidelines for animal research (e.g., the study should contribute to medical knowledge, which could ultimately contribute to human or animal well-being, and the welfare of mice should be considered as much as possible [37]). These observations may also be applicable to the transplantation of sentient human brain organoids into animals. If brain organoids are sentient (i.e., have the capacity to feel pain and pleasure), then it would be more crucial to rigorously examine (1) the purpose of the transplantation, (2) whether it can reasonably be expected to yield relevant and useful information, and (3) the welfare of sentient human brain organoids (and host animals).^12^

Further, human brain organoids with non-valenced experiences provide another issue. If the transplantation of sentient brain organoids into animals cannot be ethically justified, what about brain organoids with only a non-valenced sensory experience? The moral importance of non-valenced experience has rarely been discussed for brain organoids *in vitro* [19] and not at all when it comes to transplantation.^13^ If non-valenced consciousness is morally important, it may be necessary to reduce the number of human brain organoids used.

As mentioned above, transplantation of sophisticated human brain organoids into animals would be an important step toward future human brain organoid-to-human transplantation. Therefore, scrutinizing the moral status of various types of conscious human brain organoids is an important issue that must be addressed before transplantation into humans becomes a reality.

## Pre-Integration

### The Distinction Between Pre-Integration, Integration, and Post-Integration Stages

Next, we proceed to the phase in which the transplantation procedure is completed and human brain organoids have been engrafted in the host brain. We divide this phase into three stages: Pre-Integration, Integration, and Post-Integration.^14^

#### Pre-Integration stage

Human brain organoids cultured *in vitro* have no blood vessels, which limits the supply of nutrients and oxygen to their interior and hinders their maturation. However, when transplanted into animal brains, host blood vessels grow into them, further promoting their maturation [1]. Nevertheless, we can conclude that brain organoids in this state are not initially *functionally* integrated into the host brain. In other words, they are not functional parts of the host brain neural network.^15^ In this Pre-Integration stage, the transplanted human brain organoid and the host brain coexist in the skull in a functionally independent manner. Here, the transplanted human brain organoid is not a part of the host brain, functionally. Therefore, ethical issues regarding brain organoid welfare, moral status, and mental capacity at the Pre-Integration stage are similar to those concerning *in vitro* culture (**3**), although further maturation may make these issues more pressing.

#### Integration stage

Transplanted human brain organoids are functionally integrated, gradually, into the host brain. During this stage, special problems arise when the transplanted human brain organoids are conscious (see "Integration").

#### Post-Integration stage

In the Post-Integration stage, transplanted human brain organoids become fully functionally integrated with the host brain. The human brain organoid at this stage is significantly different from that at the Pre-Integration stage. It obtains new inputs and produces new outputs and may have acquired advanced capacities than those before. This gives rise to new ethical problems (see "Post-Integration").

The remainder of this section examines the possible ethical issues in the Pre-Integration stage.

### Possible De-enhancement in Animals

When human brain organoids are transplanted into host animals, the first problem is de-enhancement (i.e., the deterioration of the various capacities that the host animals originally had) and a decrease in the welfare of the host animal. Transplantation of human brain organoids requires the creation of a cavity and placement of a foreign body in the host brain after surgery. This remains true whether the transplanted organoid is conscious (**6**) or not (**8**). It is possible that the capacity of the host animal will deteriorate to some degree and that its welfare will be compromised. Indeed, in the first case of human brain organoid transplantation, mice performed worse in spatial memory tasks after surgery [1]. This is undesirable for aspects of animal welfare.

De-enhancement and decreased welfare are well-known ethical issues that are common to all invasive animal experiments [38]. For human brain organoid transplantation into animals, the welfare of the host animal must be given due consideration (both now and in the future). A recent study utilized the principles of animal ethics to analyze two cases of human brain organoid transplantation in animals and concluded that such transplantations would be morally inappropriate [7].^16^ It also pointed out that the behavior of chimeric animals has not been sufficiently examined to fully assess their welfare. A more rigorous assessment of the behavior and physiology of chimeric animals is needed, and further considerations for their well-being may follow. In particular, a more realistic prospect of de-enhancement will be in research that destroys a part of the animal’s brain, for example, in a monkey, to cause a severe stroke and then introduces the human brain organoid to see if it can repair the deficit.^17^ However, such research involves extreme harm to the animal, which must be balanced by the ethics of the research, particularly the necessity and probability of a large expected benefit.

It is intriguing that the recent human brain organoid transplantation mentioned in the introduction of this paper reported no behavioral or physiological changes in the host rat compared with the control. If the negative impact on the welfare of the animals involved can be reduced, this would have two types of ethical implications. First, significant improvements would be achieved in the welfare of chimeric animals. Second, this would also mean that a step toward enhancing the capacity of chimeric animals has been taken. This issue is discussed in "Enhancement and Elevation of Moral Status of Chimeric Animals".

### Conscious Human Brain Organoids *in vivo*

If a nonconscious human brain organoid transplanted into a host brain remains non-conscious (**7)**, there would be no unique ethical concerns about its welfare, moral status, or mental capacities. However, they may mature *in vivo* and become conscious (**9**). Note that this is *not* a situation in which the transplanted human brain organoid becomes functionally integrated with the host brain and achieves consciousness by obtaining inputs and producing outputs (**14**). As is the case *in vitro*, human brain organoids in the Pre-Integration stage lack inputs and outputs. Nonetheless, even without inputs or outputs, they could have “islands of awareness,” a stream of consciousness that is not formed by sensory inputs from the outside world or the body and is not expressed by motor output [40]. This possibility has been discussed for brain organoids *in vitro*; however, we believe that it may be more feasible for human brain organoids *in vivo*. As noted above, transplanted human brain organoids can be supplied with nutrients and oxygen from host blood vessels and may mature further than they would *in vitro*.

There are at least two ethical issues associated with brain organoids becoming conscious *in vivo*. The first concerns welfare, moral status, and mental capacity of conscious brain organoids *in vivo*. The second concerns human conduct: is it acceptable to allow human brain organoids to become conscious *in vivo*? Therefore, we consider these issues below.

There has been widespread concern that *in vitro* human brain organoids can be conscious and, therefore, subjects of welfare. These brain organoids should be considered based on their moral status. However, this does not necessarily mean that the creation of conscious brain organoids was prohibited. For the sake of argument, it can be assumed that human brain organoids have the same forms of consciousness and moral status as rats. Even if this happens, research using such organoids would be justifiable, given due care and sufficient scientific necessity, similar to current animal experiments [16, 34]. This presupposes assessments of (1) the scientific necessity of transplant research and (2) the consciousness and welfare of transplanted human brain organoids. The latter is challenging, but several proposals have been made. These include measurement methods based on a certain theory of consciousness, such as the integrated information theory [41], and a precautionary approach embracing the uncertainty of consciousness, where if there is a reasonable disagreement about whether the brain organoids are conscious, we should assume that they are conscious [42, 43].

Given that human brain organoids mature better *in vivo*, various concerns regarding consciousness may be the most pressing regarding transplanted brain organoids, assuming that the internal structure of transplanted brain organoids does not collapse *in vivo*.^18^ In particular, it may be possible that transplanted brain organoids could *unintentionally* mature to a stage of a higher moral status, such as that of sentient animals (i.e., animals with the capacity to feel pain and pleasure).^19^ This possibility would increase when the transplants are in large mammals because the transplanted brain organoids could acquire a greater volume and thus a greater computational capacity [4]. Before such transplants are performed, the function of the brain organoids should be thoroughly assessed when transplanted into smaller animals, such as rats.

### Making Human Brain Organoids Conscious *in vivo*

The second issue related to conscious brain organoids *in vivo* is whether brain organoids can be allowed to become conscious *in vivo* (**10**)? To clarify whether this is morally salient, we compared it with transplantation of human brain organoids that were already conscious *in vitro* (**5**).

For the sake of argument, it can be assumed that the transplantation of conscious human brain organoids into animals is morally (at least *pro tanto*) wrong, given the moral status of those organoids (see "Transplantation of Conscious Human Brain Organoids"). If so, is it not equally wrong to transplant nonconscious brain organoids into animals while reasonably foreseeing that they become conscious *in vivo*? The human brain organoid would acquire some moral status only when it becomes conscious. Therefore, the question is whether it is acceptable to create an entity with a moral status that is compromised *from the outset*.

To examine this, it may be helpful to consider relevant animal research. The creation of animals whose moral status may be compromised from the outset for scientific research is generally seen as justifiable under proper ethical codes (the most common example would be the genetically engineered mouse model of human diseases. These animals are born dysfunctional because of human intervention, and in this respect, their moral status seems to have been compromised from the outset). Thus, if the moral status of conscious brain organoids is approximately the same as that of animals, making human brain organoids conscious *in vivo* can presumably be equally justified only if the purpose and relevance of the research are reasonable and the treatments of the human brain organoids and host animals are appropriate, as seen in "Transplantation of Conscious Human Brain Organoids".^20^

## Integration

The process of functional integration of nonconscious brain organoids with the host brain (**11**) does not appear to raise any ethical problems different from those arising when transplanting other human cells into animal brains. However, the welfare, moral status, and mental capacity of chimeric animals or organoids after functional integration (**13, 14**) can raise ethical concerns (see "Post-Integration").

In contrast, the functional integration of conscious brain organoids with the host animal brain (**12**) raises metaphysical and ethical issues. Notably, it is entirely unclear what will happen in this process regarding consciousness or psychology, rather than physiology [15]. This issue is metaphysical in contrast to the other issues we have discussed, but we would like to briefly discuss it for the sake of comprehensiveness.

In the Pre-Integration stage of the transplantation of conscious human brain organoids into animals, there are two subjects of consciousness: one in the host animals (**8**) and another in the transplanted brain organoids (**9**). In the integration process, will one disappear and the other be “expanded”? If so, which will disappear? Alternatively, should we assume that both disappear and a completely different subject of consciousness emerges? Or, is it possible for the two subjects of consciousness to coexist (see "Integrated Human Brain Organoids")?

While this is an interesting metaphysical issue, it is important to emphasize that, ethically, not much depends on it. As long as the moral status of conscious human brain organoids is comparable to that of animals, their functional integration with the host brain, regardless of its metaphysical nature, could ultimately be justified with proper ethical considerations, similar to how lethal experimentation on animals or killing them for food is justified.

However, the specific forms of relevant considerations vary depending on what occurs during the integration process. Consider the following:

1. During the integration process, the welfare status of some participants may have changed. For example, the process of functionally integrating a sentient human brain organoid into the host brain may be painful. If so, pain relief may be necessary (Refinement in the principles of the 3Rs [Replacement, Reduction, and Refinement] in animal experiments).
2. Ethical problems may arise with the disappearance of a subject of consciousness *per se*. If so, pain reduction alone may not eliminate the moral wrongness of integration, and the principles of Replacement and Reduction may need to be applied more thoroughly.
3. Whether the disappearing subject of consciousness is a human brain organoid or a host animal (e.g., mouse) may be a morally important difference. While this seems explicitly speciesist, a related point is that if one of the two subjects of consciousness with different moral statuses should be extinguished, there may be some reason why we should choose one over the other. For instance, suppose tentatively that the transplanted human brain organoid has only a non-valenced experience and that its moral status is lower than that of the host mouse with sentience (i.e., the capacity to feel pain and pleasure) and normal cognitive functions. In this case, it may be argued that there are moral reasons why the mice should be preserved. Then, for example, maintaining the proportion of human brain organoids in the entire chimeric brain below a certain level may be necessary (assuming that the volume of neurons determines which subject of consciousness disappears).

Again, these issues are vague, metaphysical in nature, and of no pressing importance at present, given that conscious brain organoids are unlikely to be realized in the near future. We would encourage researchers, including metaphysicians, to further examine these issues before conscious brain organoids become a reality in the distant future.

## Post-Integration

### Enhancement and Elevation of Moral Status of Chimeric Animals

Transplanted human brain organoids can be functionally integrated into the host brain (**13**). In this Post-Integration stage, the most frequently noted concerns about human brain organoid transplantation in animals are the enhancement of chimeric animals and elevation of their moral status [5, 6, 11, 14–16, 21, 33, 34, 45–47]. The functional integration of a human brain organoid with a host brain may result in the chimeric animal gaining enhanced or even new capacities. This could, in turn, increase its moral status and, thus, increase the need for animal protection. It has been reported that mice transplanted with human glial progenitor cells perform better in multiple learning tasks [48]. Future transplantation of human brain organoids may more likely enhance animal capacites than transplantation of other human cells.

Thus, as human brain organoid transplantation into animals develops further and the enhancement of animals becomes more realistic, it is important to assess the capacities and conditions of chimeric animals more carefully. Such assessments must be fine-grained and focus on individual mental capacities which are considered morally important [5, 34]. For example, a mirror task has been proposed to assess self-awareness in chimeric animals with human brain organoids [5]. Self-awareness is morally important; however, other forms of consciousness can also be related to the moral status and/or welfare. The moral importance of mental features not usually called “consciousness” should also not be overlooked. According to recent research on animal ethics, it is not enough to consider only negative experiences of animals, such as pain and suffering [38]. An animal’s needs, capacities, or activities may be of value for protection, independent of the experiences that accompany them [20]. If so, enhancing basic mental capacities (such as those for perception, behavior, and learning) can have ethical implications for the treatment of chimeric animals. If these capacities are enhanced, the rearing environment of chimeric animals may need to be improved to exercise them sufficiently. We encourage ethicists to apply their considerations of the moral importance of a wide variety of mental features to human brain organoid transplantation in animals.

### Integrated Human Brain Organoids

Next, we examine transplanted human brain organoids in the Post-Integration stage (**14**). There have been concerns that human brain organoids may acquire inputs and outputs from host animals, leading them to acquire a more sophisticated form of consciousness [12, 14–16]. Indeed, if this occurs, we can anticipate that severe restrictions will be imposed on the creation and use of such brain organoids.

However, an important but often-overlooked question arising from the functional integration of a human brain organoid is whether it can be *an independent subject* of consciousness in the Post-Integration stage. If functionally integrated with the host brain, it may be a part of the neural network that realizes the host animal’s consciousness. In fact, this appears to be derived from several theories of consciousness. The integrated information theory claims that the subject of phenomenal consciousness is a system in which the amount of integrated information is locally maximized. The parts of that system can contribute to the content of consciousness but are not the subjects of consciousness [49]. Thus, if a human brain organoid is functionally well integrated with the host brain, the chimeric brain *as a whole* can realize phenomenal consciousness. Phenomenal consciousness of the human brain organoid alone no longer exists. The same seems true for mental properties other than phenomenal consciousness. If two neural systems are functionally integrated, how can they realize two independent consciousness?

If a functionally integrated human brain organoid is not an independent subject of consciousness, there are no ethical issues associated with consciousness. Whether this is really the case is partly a matter of scientific inquiry relating to how the transplanted human brain organoid and the host brain interact, but it is also partly a metaphysical issue relating to the relationship between the two subjects of consciousness. This is an important challenge that scientists, ethicists, and philosophers should work together to address in the future.

## Use of Chimeric Animals

In addition to the creation of chimeric animals, their use in research can raise ethical issues. The research will then be restricted depending on the acquired mental capacities and moral status of the chimeric animals [50].

### Chimeras in Pre-Integration Stage

For the use of chimeric animals implanted with non-conscious brain organoids (**15**), the ethical issues to be considered will not differ from those of other neurological chimeras. However, the situation becomes more complicated when the transplanted human brain organoids are conscious (**16**). Given that the conscious brain organoid has a certain moral status, there are *two* entities with moral statuses: the chimeric animal and the transplanted brain organoid. If transplanted brain organoids are only sentient or have a non-valenced experience, their moral status will be equal to or lower than that of a typical laboratory animal. Therefore, it is unlikely that any new type of restriction will be necessary. Nevertheless, the number of entities with moral status are important. At the very least, the Reduction and Replacement of animals or human brain organoids are needed.

The most significant concern is whether the transplanted human brain organoid has a higher moral status than the host animal. If it has, experiments that cause great suffering to the chimeric animal, or kill it after the experiment, may not be morally justified because of the moral status of the transplanted human brain organoids.^21^ Although questionable that it is justifiable to create such a chimera in the first place, transplanted human brain organoids may unintentionally reach such high levels of moral status. Again, this provides a reason to be cautious about transplantation, especially in large experimental animals, in which transplanted human brain organoids may more easily realize consciousness than they would within the brains of smaller animals.

### Chimeras in Post-Integration Stage

There are at least two additional points to note regarding the use of chimeric animals at the Post-Integration stage (**17**).

1. Because enhancement is more likely to occur at this stage, concerns about the welfare and moral status of chimeric animals may become more pressing.
2. If a functionally integrated human brain organoid does not qualify as an independent subject of consciousness and thus of welfare, consideration for the transplanted human brain organoid itself will no longer be necessary.

## Conclusion

We mapped various ethical issues related to human brain organoid transplantation into animals onto 17 states/processes in 7 stages of transplantation studies. We conclude by recapping the issues discussed in two ways.

First, our focus on transplanted human brain organoids highlights the ethical issues specific to the transplantation of human brain organoids into animals. These include:

1. Cell Collection:

- Dual sensitivities: the creation of chimeric animals and human brain organoids (**1**)

2) Transplantation:

- Transplantation into animals may compromise the moral status of conscious human brain organoids (**5**).

3) Pre-Integration:

- Concern for the welfare of transplanted human brain organoids which become conscious before functional integration with the host brain is necessary (**9**).
- Permissibility of making non-conscious human brain organoids conscious *in vivo* before functional integration with the host brain is involved (**10**).

4) Integration:

- It is unclear what happens to the consciousness of host animals and human brain organoids (**12**).

5) Post-Integration:

- Concern for the welfare of the functionally integrated conscious human brain organoid is necessary (**14**).
- Functionally integrated human brain organoids may not be an independent subject of consciousness (**14**).

6) Use of Chimeras:

- The dual presence of entities with a moral status in a chimeric animal can occur (**16**, **17**).
- The moral status of the transplanted human brain organoids may exceed that of host animals (**16**, **17**).

However, this list is by no means comprehensive. We hope that this overview will serve as the first step toward identifying issues specific to human brain organoid transplantation in animals. Probably, many of such specific issues would assume that human brain organoids are conscious. However, it is doubtful that currently used human brain organoids are conscious, and it is also doubtful if this may be possible in the near future.

Second, therefore, it would be helpful to mention some of the most pressing current or future issues related to animal transplantation of human brain organoids. These include:

1. Cell Collection:

- Dual sensitivity: creation of chimeric animals and human brain organoids (**1**)

2) Transplantation:

- Social acceptance of the disturbance of “species boundaries” (**4**, **5**)

3) Pre-Integration:

- The welfare of chimeric animals (de-enhancement) (**6**, **8**)

4) Post-Integration:

- The welfare of chimeric animals (enhancement) (**13**)

5) Use of Chimeras:

- The moral status of enhanced chimeric animals (**16**, **17**)

These pressing moral issues are common to creation of other (neurological) chimeras. Thus, no additional restrictions on human brain organoid transplantation in animals will be necessary in the near future. However, as noted throughout this paper, much remains to be achieved to prepare for the ethical assessment of future research. This includes (1) understanding citizens’ perspectives on the transplantation of human brain organoids; (2) determining the exact level of moral status of an entity that makes its transplantation into animals unacceptable; (3) calling for a more rigorous assessment of the purposes and scientific necessity of the research, and physiology, behaviors, and psychology of chimeric animals; (4) inventing methods for assessing the consciousness of brain organoids and the capacities of chimeric animals; (5) re-examining the moral importance of different mental features; and (6) tackling metaphysical problems related to the fusion of consciousness. In summary, we should take advantage of the good fortune that there is no immediate need to regard human brain organoid transplantation in animals as particularly problematic and prepare for future ethical concerns.

## Footnotes

1 For a similar analysis, see also [6].

2 Recently, some organoid researchers have suggested that the term "chimeric animals" should be avoided in relation to human brain organoid transplantation into animals [8]. Indeed, it is an important issue that many misleading terms are used in relation to brain organoid research. However, for the following reasons, we use this term in this paper. First, there is no concise and appropriate alternative term to refer to animals transplanted with human brain organoids. Second, the term has been used broadly in ethical discussions about relevant studies. Third, institutions like the International Society for Stem Cell Research and US National Academies of Sciences, Engineering, and Medicine still use it [9, 10].

3 In this paper, we use the term “integration” to refer the state in which the transplanted brain organoid is integrated into the host brain *in terms of neural function*. In other words, the term is used only when the transplanted brain organoid becomes a functional part of the host brain’s neural network. Although transplanted brain organoids also make connections to host blood vessels and other tissues, these connections are not referred to as “integration.” To emphasize this, we often add “functional” to “integration.” This point is important for the distinction between the three stages of the research we postulate (Pre-Integration, Integration, and Post-Integration). For this distinction, see "The Distinction Between Pre-Integration, Integration, and Post-Integration Stages".

4 Two studies consider several stages of the research process [14, 15].

5 For a moral evaluation of research on human brain organoid-associated sentience and cognitive advances, see [16]. There is controversy as to whether non-valenced experience also deserves protection [17, 18] (see also [19]). In addition, it is pointed out that among the various mental features that human brain organoids may possess, it is insufficient to focus only on consciousness [20]. See also "Enhancement and Elevation of Moral Status of Chimeric Animals".

6 If the transplanted human brain organoid is functionally well integrated with the host brain, it could become *a part* of the neural network that realizes consciousness of the host animal. In this case, the transplanted organoid could contribute to the host animal's consciousness and thus be called “conscious” in a sense. However, in this paper, we use "conscious brain organoid" to mean a brain organoid that realizes consciousness *by itself*, not as a part of the whole neural network that realizes consciousness. On this point, see "Integrated Human Brain Organoids".

7 We thank one of the reviewers for this point.

8 Some have even been critical of using the concept of humanization as a moral category. J. J. Koplin, for example, argues that humanization as an ethical issue is reducible to the question of the welfare and rights of chimeric animals [28]. We basically agree with this view and, therefore, do not use this term any further.

9 In contrast, scientists seem to believe that animals transplanted with human brain organoids should not be treated differently from non-transplanted animals [30].

10 We are dealing here only with ethical issues in the Transplantation stage. The later stages raise different issues related to the transplantation of human brain organoids and other human cells.

11 For this analogy to work, transplanted brain organoids have to be an independent subject of consciousness even within host brains. As discussed below, this can be possible when the transplanted human brain organoids are not functionally fully integrated with the host brains (see "The Distinction Between Pre-Integration, Integration, and Post-Integration Stages" and "Possible De-enhancement in Animals"). However, it may impossible when these are fully integrated (see "Integrated Human Brain Organoids").

12 See [38] for various ethical principles regarding animal welfare. For the welfare of transplanted human brain organoids, see "Conscious Human Brain Organoids in vivo" and especially note 19.

13 There would be little disagreement when it comes to consciousness in the sense of having cognitive functions comparable to those of humans. If human brain organoids have such a form of consciousness, then they should presumably be given the same moral status as that of primates or human beings. In this case, Lavazza's above view becomes more convincing: transplanting such human brain organoids into animals would then violate their dignity and be difficult to be justified.

14 To our knowledge, only Lavazza calls attention to the moral importance of this kind of distinction though briefly [13]. The difference between Pre-Integration and Post-Integration stages is ambiguous. In reality, functional integration is gradual with no sharp distinction between Pre-Integration and Post-Integration stages. However, the distinction is useful for heuristic purposes.

15 On our usage of the term “integration,” see also note 3.

16 Instead of the traditional three principles (Refinement, Reduction, and Replacement), the study uses the more rigorous six principles recently proposed by [38].

17 There are already studies transplanting human brain organoids into animal models of traumatic brain injury [39].

18 We thank one of the reviewers for this point.

19 Relatedly, as far as nutritional conditions are concerned, transplanted brain organoids will have a “better” environment than organoids *in vitro*. In addition, they may even be more “autonomous” in the sense that they do not need human intervention for survival (although they are still parasitic, so to speak, on the host animal [we thank one of the reviewers for this point]). If the transplanted brain organoids are conscious, this “goodness” or “autonomy” may be so in morally relevant senses.

20 The story becomes more complex when transplanted human brain organoids have advanced cognitive functions, although it is strongly doubtful that brain organoids in the Pre-Integration stage, which have no inputs or outputs, can have these functions. As an extreme case, it can be assumed that a transplanted human brain organoid has advanced cognitive functions and its moral status is equal to that of humans. In this case, the moral issues come very close to those related to human reproduction. In the Pre-Integration stage, the brain organoid lacks an entire body [44]. In the case of humans, is it morally justifiable to allow a child to exist in this state? If not, the treatment of human brain organoids should be equivalent.

21 Alternatively, one might consider that the presence of human brain organoids in the brain can elevate the moral status of the host animal. Either way, the use of the animal would have to be more strictly regulated.
